# ‘The threads in his mind have torn’: conceptualization and treatment of mental disorders by neo-prophetic Christian healers in Accra, Ghana

**DOI:** 10.1186/s13033-018-0222-2

**Published:** 2018-07-24

**Authors:** Lily N. A. Kpobi, Leslie Swartz

**Affiliations:** Department of Psychology, Stellenbosch University, Stellenbosch, South Africa

**Keywords:** Ghana, Neo-prophetic, Pastors, Faith healing, Mental disorders

## Abstract

**Background:**

In many low- and middle-income countries, faith healing is used alongside biomedical treatment for many health problems including mental disorders. Further, Christianity in Africa has seen much transformation in recent decades with the growth of charismatic or neo-prophetic churches whose doctrines include healing, miracles and prophecies. As such, many charismatic pastors have been engaged in faith healing for many years. Such faith healers form a significant portion of the mental health workforce in these countries, partly due to the limited number of biomedically trained professionals. In this study, we sought to examine the beliefs of charismatic/neo-Pentecostal faith healers about mental disorders, as well as to examine the treatments that they employed to treat such disorders.

**Methods:**

We interviewed neo-prophetic pastors who undertook faith healing, and examined their work relating to mental disorders. Ten pastors from eight churches in the Greater Accra Region of Ghana were interviewed using semi-structured interviews.

**Results:**

The data suggest that the pastors’ conceptualization of mental illness was generally limited to psychotic disorders. Their beliefs about causation were predominantly supernatural in nature although they acknowledged that drug misuse and road traffic accidents were also potential causes. The pastors’ expectations of healing also showed different perceptions of illness chronicity. Their diagnostic and treatment methods revolved around using prayer, prayer aids such as oils and holy water, as well as spiritual counselling for patients and their caregivers. However, they were not opposed to referring patients to hospitals when deemed necessary.

**Conclusion:**

We discuss the above results with emphasis on their implications for collaboration between biomedical and alternative healing systems in Ghana. In particular, we advocate a mutual understanding of illness perspectives between biomedical practitioners and faith healers as an important component for integrating different health systems in Ghana.

## Background

In many sub-Saharan African countries, alternative healing systems exist alongside allopathic systems of care. These alternative systems, such as traditional and faith healing methods, are utilized for a wide range of conditions, including mental health care [1]. One of the reasons for the popularity and widespread use of traditional and faith healing in mental health is the perceived similarities of disease causal beliefs between the healers and their clientele [2]. However, another factor is the real shortage of trained mental health professionals in many low- and middle-income countries [3].

In Ghana, some studies have estimated that there is one psychiatrist for every 1.2 million people, as well as one mental health nurse for every 200,000 people in the population [4]. A further constraint is that a majority of these professionals are located in the urban towns of the country [4, 5]. In addition, all three public psychiatric hospitals are located along the southern coast of Ghana. Although in recent years small psychiatric units have been set up in most of the ten regional hospitals across Ghana, these are invariably located in the urban/semi-urban towns of those regions. Therefore, a large segment of the Ghanaian population has limited or no access to formal mental health professionals and services. In fact, some studies have argued that only 2% of Ghanaians requiring mental health care have access to formalized care [5–7].

On the other hand, Ae-Ngibise et al. [8] estimated that there was one traditional/faith healer for approximately every 200 people. Given this, it is further argued that the first point of call for approximately 70% of the population of people who need psychiatric care would likely be an alternative medicine practitioner such as a faith healer [8, 9].

There is limited data on national prevalence rates of actual use of traditional/faith healing systems, perhaps due to the frequent use of multiple healing systems by patients. While faith healing is popular, it is often not the only help-seeking avenue explored. Gyasi et al. [10] in their study of the use of alternative healing therapies among a cross-section of Ghanaian tertiary students, reported that approximately 89% of participants had utilized more than one form of health care (including herbal, spiritual, and biomedical) in the last 12 months. Furthermore, Ofori-Atta et al. [5], in their situation analysis of mental health services in Ghana, also reported that the use of faith and traditional healing was often unreported by patients. This was largely believed to be due to the distrust that the biomedical field was perceived to have for alternative therapies [5]. Thus, prevalence rates of the use of traditional/faith healing are difficult to estimate.

A few small-scale studies have however explored the use of traditional vs. faith healing by patients in Ghana. According to Read and Doku [11], the past 30 years have seen a shift in the religious landscape of Ghana, with the perceived role of Christian healers and prayer camps showing a significant increase. 14% more people admitted turning to Christian faith healing, in addition to or instead of biomedical therapies, rather than traditional religious healing centres as had been the case in the 1970s [11]. Christian religious healing has therefore seen an increased acceptance and use in Ghana.

In the most recent census, approximately 96% of Ghanaians self-identify as being religious, spanning Christian, Muslim and indigenous African religious beliefs [12]. Of this number, an estimated 71% classify themselves as Christians [12]. Further, 28% of the estimated percentage of Christians belong to the charismatic/neo-prophetic tradition [12]. Considering this high level of religious identification, and given the shortage of mental health professionals, calls have been made to explore the benefits of engaging faith-based organizations in care for mental illness [5, 13, 14]. The calls for collaboration came particularly because many such organizations have already been involved in providing spiritual healing to their members for years [15–17].

Many studies on alternative healing practices tend to group practitioners together, and hence overlook the nuances that may exist between the different categories of healers, particularly in those which are based on faith. In addition, for religions which are not considered indigenous to the people, cultural values and practices may influence how that religion is understood and expressed. Therefore, the practices of faith-based healing systems will be influenced by both religious and cultural factors. In this paper, we focus on the perspectives of Christian healers in Ghana.

### Faith healing and the neo-Pentecostal/charismatic Christian movement

The spiritual healing provided by Christian faith-based organizations is largely carried out by those of the neo-Pentecostal/charismatic tradition. The neo-Pentecostal/charismatic Christian theology (also sometimes referred to as the neo-prophetic movement) is built on the experience of the Holy Spirit and its gifts such as prophecy, miracles and speaking in tongues [18]. There is also a distinct emphasis on success and prosperity, and as such, any illness or misfortune is often attributed to spiritual efforts targeted at preventing the achievement of those goals [19]. Therefore, much of the healing provided by charismatic Christian organizations focuses on re-establishing a balance between the individual’s corporeal life and their spiritual life in order to achieve the desired prosperity [17]. According to Asamoah [20], the neo-Pentecostal Christian doctrine differs from the doctrine of initial African Indigenous Churches (AICs) in that, the latter’s doctrine overtly incorporates elements and practices of indigenous African religious belief whereas the former rejects indigenous cultural practices as demonic, but is built on the framework of an African worldview.

As an illustration of this difference, Gifford [21] compared the charismatic churches’ emphasis on cosmological balance to ideologies which are dominant in indigenous African religious thought. However, instead of ancestral spirits and deities working in them (as is common in African traditional religion), the charismatic healers believe the healing is done through the Holy Spirit working through them [22]. Thus, the neo-prophetic churches have elements of African beliefs in their doctrine [19, 20], yet are different from the AICs’ syncretic fusion of traditional religious practices into their mode of worship. This influence is reflected in beliefs about the causes and impacts of illness and disorders, as well as their approach to healing.

According to Omenyo and Arthur [23], the methods employed during faith healing include prayers, fasting, deliverance/exorcism, as well as spiritual directives of required behavior. These processes are carried out through the laying on of hands, sprinkling of holy water, as well as anointing oils and incense. These form prayer aids for the healing process, with the ultimate aim of expelling or banishing the evil spirits which are perceived as preventing the success of the patient and/or his family [19]. ‘Exorcism’ thus refers to the process of removing evil spirits which are believed to possess and/or attack people and cause illness or misfortune [24].

Thus, neo-prophetic Christian faith healers employ specific methods in treating their patients. In order to achieve successful collaboration between the various health care systems in Ghana, the different elements of the work of faith healers must be contextually examined and understood. This includes obtaining more knowledge on the beliefs, methods, and perceived impact of mental disorders. Against this backdrop, the aim of this paper is to examine the beliefs of charismatic Christian faith healers about mental disorders and their perceived effects, as well as to describe their treatment methods, as a contribution to the discourse on finding holistic collaborative care for mental disorders in Ghana.

## Methods

### Research design

In this study, we used a qualitative approach to answer the question: “How do Christian faith healers in Accra understand and treat mental disorders?” We examined the lived experiences of the healers in treating mental disorders through an experiential qualitative design [25]. This design was useful for exploring the participants’ views on mental disorders based on their own experiences.

### Research setting and participants

The data reported in this paper are part of a larger study of different categories of traditional/faith healers in the Greater Accra Region of Ghana, which is a small coastal region in the south of Ghana. The Greater Accra Region is home to the nation’s capital city, Accra. It is cosmopolitan in nature, with a large number of inhabitants having migrated from other parts of the country. The region also has smaller rural and urban settlements on the outskirts of the region which are predominantly inhabited by indigenes of the region.

The participants for this study were pastors and leaders from self-styled Charismatic churches within and around the Greater Accra Region. These churches often hold healing services where people with all manner of ailments, including mental disorders, are treated. Ten pastors from eight churches were interviewed through individual semi-structured interview questions until data was deemed to be saturated. Although all eight churches were in the Greater Accra region, two of the participants had prayer camps located outside the region. A prayer camp is a facility run by a faith organization where sick people seeking spiritual treatment can be housed [16]. Various healing activities and programs are conducted at these camps for the patients who come there. Three of our participants were therefore pastors who sometimes performed their healing work at these prayer camps. In this study, we use the terms ‘charismatic healers’ or ‘neo-prophetic healers’ to refer to healers working within neo-Pentecostal/charismatic churches.

The churches were included in our study if they identified as neo-prophetic or charismatic in their style of worship. The pastors from these churches also needed to have worked as faith healers for at least 5 years, and be able to speak English, Ga or Twi (these are the most commonly used languages in the region).

We conducted semi-structured interviews with eight male and two female pastors/healers. Their ages ranged from 31 to 66 years with a mean age of 44.5 years. The number of years they had practiced ranged from 7 to 41 years. In the results section below, the participants are described with the titles which they used to describe themselves. Although we acknowledge that there may be different understandings of titles, we use terms such as prophets, seers, pastors, etc. as requested by the participants.

### Procedure

Following institutional ethical clearance, we approached several churches for permission to conduct the interviews. At the first three churches that we approached, the head pastors declined to participate and did not provide consent for their associate pastors to be interviewed either. They cited reasons such as conflicting schedules for this refusal, but some stated outright that they were suspicious of our true intentions. As a result of these initial difficulties, we engaged suitable gatekeepers in our subsequent recruitment efforts. These gatekeepers were members of the desired churches whom we identified beforehand to facilitate introductions to the pastors. We also used snowballing to recruit additional pastors in some cases.

The first author, who is a female, conducted all interviews, some with the help of a trained male research assistant. Both the first author and the research assistant are trained psychologists. Both are also fluent in English, Ga and Twi. Potential participants were approached, and the objectives of the study were explained to them, as well as their rights as research participants. Individual informed consent was obtained from each participant before any data were collected.

The participants were asked a range of questions pertaining to a number of different variables. Regarding their healing work for mental disorders, they were asked questions such as ‘what do you think caused the illness?’; ‘how are you able to identify what the patient’s illness is?’; ‘how would you treat [this] illness?’, and ‘how do you think this illness will affect the patient?’, among others.

All the interviews were audio-recorded once we had received verbal and written informed consent for participation and recording. The interviews took place at the workplaces of the participants. These were either in an office in the church, or in a quiet location at the prayer camps. The interviews lasted an average of 40 min and were conducted in English, Ga or Twi depending on the language that the participant was most comfortable with; in most cases, this involved a combination of English and one of the local languages.

### Data analysis

All interviews were transcribed verbatim in the language that they were conducted in. Where necessary, the local languages were translated into English, and then back translated into the local language by an independent linguist, to check for consistency and accuracy.

All data were analyzed using the ATLAS.ti qualitative data analysis software (v.8). The data were analyzed using Braun and Clarke’s [26] six-step thematic analysis guidelines. This method allows for systematic analysis of the data to unearth emerging patterns and ideas. In the first step, we familiarized ourselves with the data by reading the transcribed interviews several times. By this, we sought to understand the nature of the data and to search for the core ideas which ran through the data. Subsequently, the first author generated initial codes related to the pastors’ descriptions of their beliefs about and methods of healing mental disorders. These formed the basis for identifying patterns and meanings in the participants’ accounts. In the third step, we classified similar trends and patterns which had emerged from the data as tentative themes. The second author checked the tentative themes, and both authors discussed areas of disagreement or inconsistency. These initial classifications were then reviewed and refined by both authors in the next step, in order to properly define the themes. The revised themes that emerged from the data are presented as results below.

## Results

In this section, we discuss three themes that emerged from the participants’ accounts of their work in healing mental disorders. First, their beliefs about mental disorders. Second, we discuss their perceptions of the impact of mental disorders on the lives of their patients. Finally, we discuss the methods that they use to diagnose and treat mental disorders.

### Beliefs about mental disorders

In exploring the views that participants held about mental disorders, the predominant belief was that mental disorders resulted in what they considered strange behaviors. They all agreed that the behaviors displayed by people with mental disorders suggested a malfunction in their brains. For instance, one male participant, a 32-year-old pastor, stated the following:

*When we say mental illness, it means that the brain is not working well. Something has torn in their mind. Some of the old people will say, the threads in his mind have torn, you see? So it means that the person will be doing things that he is not supposed to do.* (P6)

Another participant described how individuals with mental disorders behave:

*We all know that the mad people…the way they behave is different from us… it is not normal like how you and I will behave. Like walking around naked, eating from rubbish dumps and so on. They are very rough…if they get angry they will go and look for a knife to come and stab the person…it is not normal.* (P8, 42-year-old male)

Apart from these, we also had descriptions about the different types of mental disorders. All the participants used the term ‘madness’ to describe what they considered as mental illness. For instance, a 41-year-old pastor described the types of mental illness as follows:

*When someone is mad, you can see it as soon as the person comes…it is something that has been added to the person and it is changing the way he behaves from a human being to, excuse me to say, an animal…for some of them, they don’t walk about naked but they will be talking to themselves… Some of them don’t eat rubbish but they will walk the whole day and you can’t tell where he is going… Some of them, they will be sitting in their corner quietly, and they look normal. But as soon as they get angry, they will start throwing things, and hitting people and stuff like that… So there are different types of madness.* (P1)

The above description was typical of all our participants. Generally, their explanations for what constituted mental illness pertained to descriptions of psychotic behavior. When we probed further with examples of other forms of common mental disorders (such as depression, anxiety, etc.), the consensus was that these were not the same as madness but could lead to that if not checked:

*No, that one is not a mental problem…but if she does not go for healing, then she can also start talking to herself or maybe even remove all her clothes!* (P5, 52-year-old prophetess)

Despite this perspective about psychotic disorders representing all mental disorders, some of the participants described different types of psychotic behavior as different disorders. For instance, one prophet explained that the different types of mental disorders were as follows:

*But we have three different types of madness. Some people…you may think that they are fine but when you trace their [speech] to a certain level, you will see that the person is communicating with you but it doesn’t make sense to your satisfaction…to give you the specific answer that you need from the person… It is like he is talking to somebody that only he can see. And then we have another group…when you are communicating with them you will see that their minds cannot focus or concentrate on anything to do it well…they always jump from one thing to another and do it shabbily… Then we have those people who are deeply sick with that sickness. Those ones you see them they will be drinking water from the gutters, they will be eating from [rubbish dumps] and all kinds of strange things… that is also another group.* (P3, 48-year-old prophet/seer)

In addition to the views on mental disorders and the different types, we also explored their beliefs about causes of the disorders. Perhaps unsurprisingly, the predominant belief was that mental disorders were caused by evil or unclean spirits and witchcraft. All ten participants shared this belief. One participant described it this way:

*Witchcraft, idol*-*worship, and family gods can all be linked to spiritual factors that can affect the person’s mental health. Especially when you’re young, and you start behaving like that then you have to start suspecting that there is a spiritual dimension that is responsible for what is happening. Because you know with us Black people, that is, we the Africans…we have all these family gods and spirits that are still around us and if they don’t like something that you are doing, they can attack you and torment you and you will never feel happy.* (P7, 39-year-old prophet)

Some of the reasons that they cited for the attack by the unclean spirits included envy or jealousy from others:

*I have recognized that some of the mental sicknesses are brought by unclean spirits from various families… when they see the future of someone, then they throw that sickness to that person…yes, because they have seen the person’s future! And they want to destroy it. They don’t want him to succeed in the future.* (P3)

Although this was the predominant view of the healers, all participants acknowledged that sometimes the disorder was not brought about by spiritual factors alone. Some of the other factors described were drug or alcohol misuse, as well as traumatic brain injury resulting from road traffic accidents. Nevertheless, they believed these other causes invariably included a spiritual dimension as well.

A third component of the healers’ beliefs was their perceptions about the chronic nature of mental disorders. To most participants, treatments were meant to ‘cure’ illness; therefore, if the patient has been taking medication for the same illness for a prolonged period, then it was ineffective. One prophet was quite emphatic in his views on this:

*How can you take medicine for one problem for the rest of your life? Have you heard of something so strange before? What kind of sickness does not go away? If the mosquito bites you, it leaves something inside your body, and then you get malaria. So the medicine that [the doctors] give you will kill that thing that the mosquito put inside you. But if you take the medicine every day for so many years and the malaria is not going [away], then it means the medicine is not good, yes…because it is not working! How can you be taking medicine for the same mental problem for so many years and it still keeps coming back?* (P9, 31-year-old pastor)

Such views obviously confirmed their beliefs about spiritual causes for mental disorders. Since they believed most mental disorders had a supernatural origin, they firmly believed that they needed to be involved in its treatment. All the participants believed that once the illness that afflicted a patient was spiritual, biomedical methods would always be ineffective in the long term because doctors were not equipped to treat spiritual problems. As such, the emphasis was very much on making sure that the symptoms disappeared completely, which was taken as an indication that the treatment was efficacious because the patient had been cured.

### Perceived impact of mental disorders

Considering the strong emphasis on spiritual causation of disorders, we also explored the participants’ perceptions of how living with a mental disorder affected the lives of their patients. All the participants believed that nobody should have to experience living with a mental disorder because it was damaging to their livelihood and to their future:

*Such a sickness…it won’t allow you to progress in life… Your goals will be delayed, and you lose your glory and pride in life… You can also lose your friends and even your money. In fact, you can lose everything because you will be looking for answers and so you won’t be able to work… And if you can’t eat or sleep, you will lose weight… your health will also decline and so on. So you won’t be happy, and you know, every human being needs to feel happy for you to live well.* (P4, 56-year-old prophet)

*It makes the person die early. He loses his school or his work, and in fact, everything in their life because of the way the [illness] comes upon them. In fact, they lose the joy of life when they have this illness.* (P10, 39-year-old prophetess)

Some of the participants also made statements suggesting the deep stigma that they believed was attached to living with a mental disorder:

*Hmmm, this illness it can destroy your life, oh! Because she won’t be able to do anything normally, so her life will definitely be difficult… And many people don’t want to marry someone who is mad…even if someone in your family is mad, nobody wants to go to that family. Because, excuse me to say, she is a human being but she is not one of us…* (P6)

Thus, there is a lot of stigma attached to having a mental disorder and this stigma extends, by association, to members of the patient’s family. Despite such apparently negative views, all the participants believed firmly that these disorders could be healed and the associated difficulties and stigma could disappear.

This relates to supernatural beliefs, particularly those regarding attacks from jealous or envious people. Paradoxically, their accounts suggest that since such conditions are the result of malicious spiritual attacks, the patient bore no responsibility for having this illness. This may very well be a means of addressing the stigmatizing nature of living with (or having a relative who has) a mental disorder, by believing that the illness could be cured by a higher power.

### Diagnosis and treatment of mental disorders

A third theme that emerged related to the methods that the pastors used to diagnose and treat mental disorders. What quickly became clear was the fact that healing depended on the abilities of the pastor. Some of our participants spoke about the different types of pastors that existed and the level of divine anointing that each category possessed. This anointing determined the success of their methods. For instance, one of the participants explained the categories in this way:

*We the prophets, we have three different categories when it comes to the office of prophecy. We have the minor prophets, we have the major prophets, and then we have the seers. The minor ones are the ones who are now up and coming; they see things but they don’t see too far spiritually. And then, as to the major ones, they see it, speak to God and speak the mind of God to people, and through the word of God that is revealed to him, they can heal some people but not all. But then the seers, when they see someone, they will be able to identify the kind of problem that person has immediately, and once they speak, every sickness on this earth will flee. I am beyond the major prophets; I am a seer. I am on the mantle with God. I see God face*-*to*-*face, Jesus is my friend, and I move with the armies of God. Do you understand? It is not a simple thing…it depends on how deep someone is able to align himself with the gift that God has given to that person. So when I see people, I am able to see what the problem is; then I discern spiritually how to heal the person.* (P3)

All the participants stated that their treatments always started with some preliminary investigations of the symptoms or behavior of the patient. These preliminary investigations included interviewing the patient and/or their family, observations of their behavior, and sometimes physical examinations of the patient’s body:

*When they come, I have to first interview them, or if the mind is not stable, then I will have to ask the relatives why they have brought him to me. I will ask them when it started, and if it is on and off, or if it has been happening continuously since it started…then I will also do my own observation of the person for some time.* (P5)

All the participants also stated that they used prayer to identify the disorder and its causal factors, as well as to treat the problem. However, what became evident was the fact that prayer involved much more than verbal supplication. They used the blanket term prayer to refer to a range of activities whose ultimate aim was to exorcise the demon from the patient. Further, exorcism appeared to be used to deliver individuals who were believed to be possessed by evil spirits, as well as to ward off the evil spirits which attacked and tormented people. The exorcism activities included issuing verbal commands (sometimes referred to as ‘binding and loosing’), dreams, and speaking in tongues. All these were divination methods that were used to identify the spirit causing the problem in order to remove its influence:

*When they bring [a patient] to me, maybe the person has been hearing voices in his head, or he will be hearing that someone is commanding him… So when they come, I first have to pray over the person using the tongues, and sometimes I even have to ask the people to go and come back the next day so that maybe I will ask God to speak to me in a dream about the issue. And when I am praying I ask God to show me who is causing the problem and also how I am supposed to treat this.* (P4)

Some others credited their healing ability to the special gift that they had been given by God:

*Everything that I do here, it is God who reveals it to me. Everything depends on how God uses someone. When we speak in tongues, we are able to ascend to the throne of God and he reveals to us everything that we are supposed to know about the case… It is not easy to heal the mentally ill people… But I have been healing people not by my might, not by my power, but by the spirit of the most high God.* (P3)

*You see, God has given us the power to do wonders. The Bible says that whatever we bind on earth will be bound in heaven, and whatever we loose on earth will be loosed in heaven. So whenever they come here, once we start praying and start speaking in tongues, we can bind the demons that have caused that sickness for the person and command it to loose [its hold over] the person’s life [sic].* (P2, 66-year-old prophet)

The mode of diagnosis was therefore reliant on the healers’ God-given ability to discern the spiritual causes of the illness, and by extension, the treatment also relied on that ability. Because the method of treatment was predominantly prayer, the regimen tended to change from case to case:

*I won’t be able to tell you that for this type of case, we pray for one week or for that type of case it is intensive fasting, because everybody’s case is different. Sometimes we have to pray continuously for one month before we will see any improvement.* (P8)

Prayer was therefore used for both diagnostic purposes and for treatment purposes. Apart from prayer, the prescribed treatment sometimes included fasting by the patient:

*Sometimes the patient will have to fast before he gets his healing…the fasting means denying yourself of something so that you can elevate yourself to a higher level in the spirit… Sometimes people think fasting is all about food or that you have to starve yourself. No, it is more than that… Although fasting with food is very important, you can also fast with other things, like your time or your work… If you want to elevate yourself to another spiritual dimension, you deny yourself of that thing and then you concentrate on the spirit. But food is the strongest way to fast because it forces you to take your mind away from the physical and to focus on God.* (P1)

When questioned about a mentally ill patient’s ability to fast appropriately, some of the participants explained that other people could fast for the patient:

*Yes, if we see that his mind cannot focus on God because of the way the sickness has made him, then we can get one of his family members to fast for him, or even we the pastors can fast for him, and intercede for him.* (P5)

In addition to prayer and fasting, some of the participants spoke about using prayer aids such as anointed oils and holy water to exorcise the evil spirits:

*We don’t normally lay hands on them straightaway… we will first apply anointing oil and they will rub it all over their heads so that the brain comes back a bit…then you can sprinkle some holy water on them before you tackle the real healing.* (P6)

This participant’s account suggests that the actual healing is considered as the laying on of hands by the pastor. Others also stated that once the patient had been ‘primed’ by prayer, fasting, and the use of different prayer aids, the healing could be completed when the pastor laid his hands on them. According to some participants, this was the final act which would expel the evil spirit completely.

Another method that was used to complete the healing process was what many of the participants called ‘spiritual counseling’ or ‘spiritual directions’. This was explained to us as similar to the counseling offered by professionals but having a spiritual focus. According to the participants, when patients relapsed, it was because they had either made a mistake that had allowed the spirit to re-enter their bodies, or they had failed to adhere to some prescribed guidelines. As a result, some patients needed to receive special counseling to show them how to prevent another episode. Sometimes, these directives were also to complete the healing process, especially if they needed to make restitution for some sin. One participant explained this process to us:

*Sometimes, we have to listen to their problem and give them some counseling on how to solve it… Sometimes, they have to go and give a gift to someone… and I am told [by God] the number of people that they have to go and see, for instance they have to give something to five people or ten people or whatever… Sometimes, you have to do it at a particular time of the day, or sometimes it has to be money; sometimes you have to cook a large meal and invite people to come and eat it… So once you do these things, it forces the spirit to leave your body and then you can come back to normal… So this is an example of the direction… we have to explain everything to the person so that he can go and do it well, then the spirit causing it cannot come back again.* (P4)

Even though participants emphasized the need for spiritual healing for mental problems, they did not deny that biomedical care was also necessary. All the participants believed that patients needed to receive biomedical care for the physiological effects of their illness. They believed this could be done alongside the spiritual care that they provided. Some participants also stated that sometimes, God revealed to them that the appropriate treatment was from the hospital:

*Like I said, he can go to the hospital for treatment as well because God sometimes works through such people too… There are some things that are not for God to do himself; they must be done by people… God is the one who gave them the wisdom and the ability to know how to treat people. There is no way God will come down from heaven to come and inject anybody with any medicine the way the nurses go round and inject people. He works through people that he has given the ability to use the medicines to heal people. So if it is necessary, he can go to the hospital after he leaves the prayer camp.* (P1)

Therefore, the biomedical treatment was believed to augment the spiritual treatment in order to complete the healing. Many of the participants believed that evil spirits could cause even non-psychiatric disorders. Although caused by spiritual forces, they may manifest in physiological ways. Thus, all ill health potentially had a spiritual component.

## Discussion

In this paper, we have examined faith healing for mental disorders from a neo-prophetic Ghanaian Christian perspective. We described the pastors’ beliefs about the causes and impacts of mental disorders. We also examined their diagnostic and treatment methods.

The general perception of the participants was that evil or unclean spirits caused most mental disorders. Despite this prevailing belief, most participants acknowledged other potential causes of mental disorder such as drug or alcohol misuse. However, these were mostly seen as a moral failing on the part of the patient. Because of these beliefs, their methods of diagnosis and treatment involved activities aimed at exorcising or warding off the demons from the patient. Such methods included using prayer and prayer aids like holy water, as well as fasting, speaking in tongues and counseling. This spiritual counseling, as described by some of our participants, was strikingly similar to what has been reported as being used in traditional African shrines [27–29].

Even though our participants emphasized a spiritual focus for healing mental disorders, they were not opposed to patients receiving biomedical care in addition to the spiritual care; in fact, many reportedly frequently referred patients to hospitals. This seems contradictory to the apparent beliefs about the spiritual management of spiritual illness. Given the perceived expectations of curing sickness, it was a bit surprising that they reported referring patients to the hospital for management; especially for the treatment of illnesses that they considered to be supernatural in origin. But although they referred patients to hospitals, the expectation of a cure was still present and as such, they did not expect their patients to continue presenting with symptoms. Although there is mixed evidence in biomedical thought about recommending prolonged pharmaceutical treatment of psychotic disorders [30], our participants believed that an effective treatment of illness should not be prolonged. This is seen in the participants’ own treatment where remissions are explained as a patient’s failure to adhere to instructions. To our understanding, the participants did not seem familiar with the concept of partial remission in serious mental disorder.

The findings reported in this study are consistent with what has been reported in other studies in Ghana. For instance, Asamoah et al. [15] examined the perceptions of Pentecostal clergy in Ghana on the causes of mental disorders, among others. Like our participants, their findings suggested a strong belief in supernatural causes of mental illness, as well as the role that they needed to play in healing the mentally ill. Therefore, the dominant perception of Ghanaian clergy appears to be that mental disorders result from diabolical intentions [1, 17, 20].

This supernatural perception of illness causation is not limited to clergy alone. Yendork et al. [31] reported that congregants of charismatic churches also attributed mental illness to supernatural and diabolical forces. However, Opare-Henaku and Utsey’s [32] exploration of indigenous Akan concepts of mental illness suggests that such notions are likely influenced by cultural perceptions of illness causation, further emphasizing the syncretic nature of the neo-prophetic churches.

Again, this is not different from what has been reported in other countries. In their exploration of conceptualization of psychosis by indigenous and religious healers in Uganda, Teuton et al. [2] reported that their participants regarded mental illness as communication from family spirits, persecution by others and punishment. What was different in our study was that our participants did not view mental illness as originating from ancestral communication, although the belief that persecution and punishment could cause such disorders was present.

Unlike what has been reported in some other African studies [2, 33], unheeded ancestral communication which results in mental illness is, to our knowledge, not very common in the Ghanaian context. Instead, much emphasis has typically been placed on illness and misfortune arising due to the displeasure or punishment of gods and deities, or from witchcraft activities of malevolent persons [22, 27]. This is reportedly a feature of indigenous religious thought. Thus, the spiritual factors associated with mental illness also reflect the influence of indigenous ideas.

With regard to the methods employed, Agara et al. [34] also reported that their sample of Nigerian clergy employed methods such as prayer, fasting, and prophecy to diagnose and heal mental disorders, just as our findings suggest. However, their participants reported beating the patients as a means of banishing the evil spirit; this was different in our sample, as some of our participants stated that they did not believe in such methods. But this could very well be a matter of providing socially desirable answers by some of the participants, given that some media stories in recent times have reported that patients were being beaten in some prayer centers in Ghana [35].

These findings can also be examined with respect to neo-Pentecostal/charismatic churches in non-African countries. For instance, in a study of explanatory models of mental illness among clergy from different ethnic backgrounds in the East London area, Leavey et al. [36] reported more mixed etiological notions among their participants, including supernatural, biomedical, as well as social/situational explanations for mental disorders. The participants’ ethnic backgrounds included English, African, Caribbean, and South Asian. Although some similarities can be drawn between these beliefs and those of our participants, there was a higher endorsement of biomedical and situational explanations for mental disorders among the non-African participants in their study. Thus, the African cultural influence on Christianity is seen here also. These similarities have also been reported in studies with immigrants in other contexts [37, 38].

### Implications of our findings

These findings have varied implications. In the first place, it seems clear that the clergy strongly believed that because of the supernatural etiology of mental disorders, they needed to be part of patient care. This willingness may be a good opening for collaborative efforts, particularly given the fact that many of their congregants reportedly hold similar causal beliefs. However, it would be wrong to deny the factors which may make such partnership difficult. Foremost are the varied methods that are used for diagnosis and treatment. Their reliance on divine revelation for these purposes implies that there is a high level of subjectivity in their work. Therefore, standardizing these treatments would be difficult, and without standard procedures the potential for abuse (even inadvertent abuse) can make collaboration problematic.

The clergy also had a high level of confidence in their abilities. According to the participants, this was because they represented God. They therefore expected to be obeyed, and through such obedience, the patients would receive their healing. Their belief in the divine source of their abilities, it seems, may have led to a belief that theirs was the only legitimate way to understand disorders and to practice healing. The expectations that patients should comply with a treatment regime because of its divine origins can be worrying because it can potentially deny patients agency. Their reliance on the pastor for direction can become an unhealthy dependency, affecting their ability for personal decision-making. It may also foster cognitive dissonance in some patients when symptoms remain or recur, despite their faithful adherence to the directives given [39, 40]. All these are potential trigger points for exacerbating their conditions, or contributing to the development of other mental disorders.

Despite the beliefs in the spiritual nature of most mental disorders, the participants acknowledged that biomedical care was sometimes warranted. However, they relied on their observation of behavior changes to confirm whether healing had been completed. Therefore, patients who continued presenting with symptoms were considered unhealed and thus, the treatment was potentially ineffective. In contrast, the healers’ reliance on behavioral changes as indication of healing could suggest that patients in their care could potentially remain in that state for prolonged periods. The absence of clear timelines for treatment is concerning and can present problems in collaborative efforts.

Due to the belief in spirits causing mental illness, it appears that blame is removed from the patient to outside influences. Thus, the level of stigma attached to living with a mental disorder is reduced, because the patient is seen as a victim. However, this may cause social and familial strains because blame is always placed on someone within the patient’s circles. Despite its usefulness for removing stigma, it may create further challenges if not redirected properly.

Although patient blame may be removed with respect to initial onset, our data suggest that patients do get blamed for relapse of the illness. As was indicated in previous section, when symptoms recurred, the patients were believed to have defaulted in the prescribed treatment. This also highlights the high level of confidence in the healing process. Although perceptions of patient non-compliance are not unusual in any healing system, such beliefs are important to acknowledge as they may be triggers for dissonance, distress and potentially other conditions. The beliefs are also important to consider in biomedical interventions given the perceived pluralistic nature of mental health care in Ghana.

The clergy also appeared to hold different notions about what can be considered universal mental disorders. Their assertion that every case is different is a divergence from the idea of standard packages of care for mental health. This lends support to the idea of rethinking the emphasis on westernized models of mental health in the scaling up of services in non-western contexts [41]. This is an important point to consider when establishing pathways to collaboration between faith healing and other therapeutic systems.

The above points are not to suggest that there is no place for Christian healers in the Ghanaian mental health system. The benefits of having congruent healer-patient beliefs is known; as is the value of spirituality in illness recovery and management. Therefore, some of the difficulty in collaboration may perhaps be resolved if the role of the clergy in mental health care is reinterpreted. The pastors’ positions and their influence as leaders are potentially beneficial for partnerships with other health systems to drive public mental health education and health promotion efforts. Thus, given that their concepts of mental illness were dominated by psychotic behaviors, some training on recognizing different disorders could further help the pastors to play an important role in facilitating appropriate referrals for care. In addition, the participants’ accounts show that the pastors already do some counseling of patients. Again, this means that they are well placed to facilitate psychosocial rehabilitation and community re-integration programs. The pastors can also be valuable partners in fostering behavior change and treatment compliance for patients who require added support.

## Conclusions

In this study, we explored the perspectives of neo-prophetic Christian healers about mental disorders. The healers were heavily reliant on divine revelation as a means of diagnosing and treating mental disorders. As such, their methods varied from one patient to another. Given the perceived high patronage of these facilities [8], their use of prayer, fasting, and holy oils appears to be accepted by patients and their families.

We do not have data on the relative efficacy of faith healing versus biomedical approaches at this time (and this is clearly an important topic for further research) so we do not wish to claim here that biomedicine is definitely more efficacious than faith healing. At this stage of knowledge, and given the pattern of resources in Ghana, it is important that different systems of healing have a better understanding of one another. So if faith healers receive psychoeducation, it should be in a context in which mental health practitioners are also open to learning about faith healing. In fact, our own research, and this article are designed partly to provide such avenues for education of mental health practitioners on this issue.

In order to effectively partner with faith healers, these factors need to be carefully considered. It is noteworthy that participants’ descriptions of mental disorders were limited to severe and disruptive mental disorders, whereas most mental disorders as understood in the biomedical system are not necessarily severe or socially disruptive. This suggests that a further area for exploration would be a discussion with pastors specifically regarding common mental disorders and their care. In addition, perspectives and experiences of patients who have attended such healing services would also be helpful in determining effectiveness. Regulations must also be enhanced in order to assess quality and efficacy. All these need to be explored if collaboration between the various systems of care is to be achieved in order to transform mental health care in Ghana.

### Study limitations

This study had a few limitations which are important to acknowledge. First is the fact that the churches which were included in this study were limited to neo-prophetic/charismatic churches. Although the participating churches were chosen due to their popular healing activities, we do acknowledge that other church denominations (such as AICs or western mission churches) may have provided different perspectives on mental health. Secondly, limiting participants to those living/working in Accra certainly also influenced the perspectives that were shared. As has been described above, Accra is a peri-urban setting which may present a worldview which differs from those in rural settings. A third limitation was the fact that the first author (who is female) conducted the interviews with a largely male population. Although this was not overtly experienced, the first author’s gender may have influenced her engagement with the participants.
